# Tissue-specific chemical profiling and quantitative analysis of bioactive components of *Cinnamomum cassia* by combining laser-microdissection with UPLC-Q/TOF–MS

**DOI:** 10.1186/s13065-018-0438-x

**Published:** 2018-06-21

**Authors:** Wenwen Zhou, Zhitao Liang, Ping Li, Zhongzhen Zhao, Jun Chen

**Affiliations:** 1 State Key Laboratory of Natural Medicines, Department of Pharmacognosy School of Traditional Chinese Pharmacy, China Pharmaceutical University, Tongjiaxiang-24, Nanjing, 210009 China; 2 School of Chinese Medicine, Hong Kong Baptist University, Kowloon, Hong Kong Special Administrative Region China

**Keywords:** Essential oils, *Cinnamomum cassia* Presl, LMD, UPLC-Q/TOF–MS

## Abstract

**Background:**

Cinnamomi Cortex, the dried stem bark of *Cinnamomum cassia* Presl (*Rougui* in Chinese) has been widely used in traditional Chinese medicine, cooking and perfumery for thousands of years. Traditionally, the Cinnamomi Cortex of thick size is considered to be of good quality; however, there is no scientific data to support this point. Considering that essential oils are the main bioactive components, Cinnamomi Cortex of greater variety and amount essential oils is thought to be of better quality. In this study, laser microdissection coupled with ultra-high performance liquid chromatography-quadrupole/time-of-flight-mass spectrometry (UPLC-Q/TOF–MS) was applied to profile the essential oils in different tissues of Cinnamomi Cortex and to determine if there is a correlation between the essential oil content and the stem bark thickness.

**Results:**

We report the tissue-specific metabolic profiles of different grades of Cinnamomi Cortex. Nineteen chemical components were unequivocally or tentatively identified in the chromatogram of the test samples. The results indicate that the bioactive components, the essential oils, were mainly present in the phloem.

**Conclusion:**

Phloem thickness is the key character for evaluating the quality of Cinnamomi Cortex. Our results can be of great importance in improving the cultivation, harvesting, and processing of Cinnamomi Cortex, as well as enhancing its effects in clinical applications.

**Electronic supplementary material:**

The online version of this article (10.1186/s13065-018-0438-x) contains supplementary material, which is available to authorized users.

## Background

Cinnamomi Cortex, is the dried stem bark of *Cinnamomum cassia* Presl, known as *Rougui* in Chinese. It has been widely cultivated in Southeast Asia and is commonly used in pharmaceuticals, cooking and cosmetics. Essential oils have been proven to be the main active components of Cinnamomi Cortex [1], with cinnamaldehyde making up between 17.1 and 87.23% of these oils [2]. Coumarin, cinnamyl alcohol, cinnamic acid and 2-methoxycinnamaldehyde also comprise significant proportions of the essential oils [3]. Previous pharmacological studies have demonstrated that the essential oils of Cinnamomi Cortex have antioxidant, antidiabetic, anti-platelet aggregation and antifungal activities [4–7]. Thus, in this study, five compounds, namely coumarin, cinnamyl alcohol, cinnamic acid, cinnamaldehyde and 2-methoxycinnamaldehyde, were selected as chemical markers for determination.

Currently various specifications of different grades of Cinnamomi Cortex have been found in the herbal market, such as *Zhong tong* (cylindric as sample RGgxdxzt), *Ban gui* (plate-like as sample RGgxpnbg), and *Guan gui* (scroll-like or groove shape as sample RGgxpngg). In clinical applications, they are typically used without discrimination, but is there a clinical difference? Comparing the chemical composition of different grades will enable us to determine the difference between grades and will help us evaluate whether these differences are significant in terms of applications. Modern laboratory studies have focused on HPLC-based fingerprint chromatography and determination of characteristic components [8–10]. However, evaluating the quality of Cinnamomi Cortex by modern instruments is time-consuming and inconvenient. Traditionally, the Cinnamomi Cortex of thick size is thought to be of good quality; but there is no scientific evidence to support this point. In the present study, various samples of Cinnamomi Cortex of different grades were collected for tissue-specific chemical analysis combining laser micro-dissected system (LMD) with ultra-performance liquid chromatography quadrupole time of flight mass spectrometry (UPLC-Q/TOF–MS). Through this study, the relationship between microscopic features and active components can be established; this relationship will enable people to evaluate pharmaceutical quality of Cinnamomi Cortex by appearance. The research also provides helpful information that can guide the cultivating, collecting and processing of Cinnamomi Cortex for maximum quality in applications.

## Experiment section

### Plant materials

**Fig. 1:**
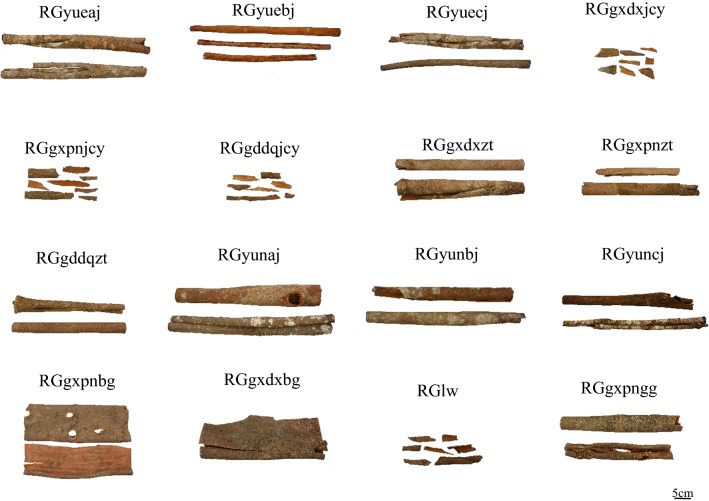
The characteristic appearance of cinnamon materials

The plant materials were collected from six major cultivation areas. Table 1 shows the details including sources and morphological descriptions for each sample. Figure 1 shows the characteristic appearance of a sample. All the plant materials were identified by Prof. Zhongzhen Zhao, School of Chinese Medicine, Hong Kong Baptist University. The voucher specimens are deposited in the Bank of China (Hong Kong) Chinese Medicines Centre of Hong Kong Baptist University.

**Table 1:** Sample information of *Cinnamomum cassia* materials

Sample no.	Locality	Grade	Morphological description		Mean thickness (mm)	Proportions of each tissue (%)			
			Surface	Cross-section		CK	C	PE	PH
RGyueaj	Wen’an, Vietnam	Grade A	Externally greyish-white, slightly rough, showing greyish-green streak, internally reddish-brown	Pericycle banded	3.7	6	13	5	76
RGyuebj	Wen’an, Vietnam	Grade B	Both externally and internally reddish-brown, slightly even	Pericycle banded	3.0	–	20	14	66
RGyuecj	Wen’an, Vietnam	Grade C	Externally greyish-brown, slightly rough, showing greyish-white streak, internally reddish-brown	Pericycle banded	3.1	6	17	11	66
RGgxdxjcy	Guangxi, China	Not specific	Externally greyish-brown, slightly rough, internally pale brown	Pericycle banded	3.1	7	24	28	41
RGgxpnjcy	Guangxi, China	Not specific	Externally brown, slightly rough, internally brownish-red	Pericycle banded	2.4	4	20	11	65
RGgddqjcy	Guangdong, China	Not specific	Externally greyish-brown, relatively rough, internally pale brownish	Pericycle banded	4.1	5	27	28	40
RGgxdxzt	Guangxi, China	Zhong tong	Externally greyish-brown, slightly rough, internally dark brown	Pericycle banded	3.7	4	29	25	42
RGgxpnzt	Guangxi, China	Zhong tong	Externally pale brown, slightly rough, internally dark brown	Pericycle scattered	5.9	5	32	38	25
RGgddqzt	Guangdong, China	Zhong tong	Externally greyish-brown, slightly rough, internally brownish-red	Pericycle scattered	4.7	10	17	24	49
RGyunaj	Yunnan, China	Grade A	Externally greyish-brown, relatively rough, showing greyish-white or greyish-green streak, internally reddish-brown	Pericycle banded	4.1	7	16	10	67
RGyunbj	Yunnan, China	Grade B	Externally greyish-brown, relatively rough, showing greyish-white or greyish-green streak, internally reddish-brown	Pericycle banded	4.3	2	21	38	39
RGyuncj	Yunnan, China	Grade C	Externally greyish-brown, relatively rough, showing greyish-white or greyish-green streak, internally reddish-brown	Pericycle scattered	3.8	5	24	26	45
RGgxpnbg	Guangxi, China	Ban gui	Externally dark brown, slightly rough, internally brownish-red	Pericycle banded	6.0	6	31	21	42
RGgxdxbg	Guangxi, China	Ban gui	Externally greyish-brown, slightly rough, internally dark brownish-red	Pericycle scattered	2.4	5	31	29	35
RGlw	Laos	Not specific	Externally greyish-brown, slightly rough, internally dark brown	Pericycle banded	3.0	6	27	34	33
RGgxpngg	Guangxi, China	Guan gui	Externally dark brown, slightly rough, internally pale brown	Pericycle banded	3.6	4	55	16	25

### Chemicals and reagents

Chemical standards including coumarin, cinnamyl alcohol, cinnamic acid, cinnamaldehyde and 2-methoxycinnamaldehyde were purchased from Shanghai Tauto Biotech Company (Shanghai, China). The purity of each standard was over 98%. Acetonitrile and methanol of HPLC grade were from E. Merck (Darmstadt, Germany), and formic acid of HPLC grade was from Tedia (Fairfield, USA). Water was purified using a Milli-Q water system (Millipore; Bedford, MA, USA).

### Materials and instruments

Leica Laser microdissection 7000 system (Leica, Benshein, Germany), Agilent 6540 ultra-performance liquid chromatography quadrupole time of flight spectrometer equipped with a mass hunter workstation software (Agilent version B.06.00 series, Agilent Technologies, USA), Cryotome (Thermo Shandon As620 Cryotome, Cheshire, UK), Ultrasonic instrument (CREST 1875HTAG Ultrasonic Processor, CREST, Trenton, NJ), Centrifuge (Centrifuge 5417R, Eppendorf, Hamburg, Germany), Electronic balance (Mettler Toledo MT5 style), Nonfluorescent polyethylene terephthalate (PET) microscope steel frame slide (76 × 26 mm, 1.4 μm, Leica Microsystems, Bensheim, Germany), Centrifuge tube (500 μL, 1.5 mL, Leica), HPLC grade vial (1.5 mL, Grace, Hong Kong), glass insert with plastic bottom spring (400 μL, Grace, Hong Kong), Acquity UPLC BEH C18 column (2.1 × 100 mm, 1.7 μm, Waters, USA), C18 pre-column (2.1 × 5 mm, 1.7 μm, Waters, USA).

### Sample solution preparations

The dried medicinal materials were firstly softened by infiltrating with water-soaked paper. The softened Cinnamomi Cortex was cut into small sections, fixed by cryogen, and then frozen on a − 20 °C cryobar. Serial slices of 40 μm in thickness were cut at − 10 °C. Each cross-section of tissue was mounted directly to a non-fluorescent polyethylene terephthalate. The slide was exposed under a Leica LMD 7000 microscopic system. Microdissection was conducted by a DPSS laser beam at 349 nm wavelength, aperture of 30, speed of 3, power of 50 μJ and pulse frequency of 1695 Hz under a Leica LMD system at 6.3 × magnification. Four different target tissues, approximately 1 × 10^6^ μm^2^ per each, were individually separated. The microdissected tissues fell into caps of 500 μL micro centrifuge tubes by gravity. Lastly, the separated tissue part in each cap was transferred to the bottom of the tube by centrifuging for 10 min (12,000 rpm, 17 °C). 100 μL methanol was added into each micro centrifuge tube. The tube was sonicated for 60 min and then centrifuged again for 10 min (12,000 rpm, 17 °C). 90 μL of the supernatant was transferred into a glass insert with plastic bottom spring in a 1.5 mL brown HPLC grade vial and stored at 4 °C before analysis.

### Standard solution preparation

Each standard compound was accurately weighed by an analytical balance and dissolved in methanol to produce mixed stock solution with concentrations at 103.05 μg/mL of coumarin, 12.32 μg/mL of cinnamyl alcohol, 132.7 μg/mL of cinnamic acid, 106.94 μg/mL of cinnamaldehyde, 157.6 μg/mL of 2-methoxycinnamaldehyde. A series of mixed standard solutions was prepared by dilution with methanol.

### Method of UPLC-Q/TOF–MS

The UPLC-Q/TOF–MS analysis was conducted at room temperature (20 °C). The mobile phase consisted of 0.1% formic acid–water (A) and 0.1% formic acid-acetonitrile (B). The gradient program was optimized as follows: 0–8 min, 5–35%B; 8–21 min, 35–65%B; 21–27 min, 65–100%B; 27–31 min, 100%B; 31–31.1 min, 100–5%B; 31.1–35 min, 5%B. The injection volume was 3 μL for each sample. The flow rate was set at 0.4 mL/min. The mass spectra was acquired in positive mode with mass to charge ratio (m/z) ranging from 100 to 1700. The operation parameters of the mass spectrometer were set as follows: dry gas temperature, 300 °C; dry gas (N_2_) flow rate, 8.0 L/min; nebulizer pressure, 40 psi; capillary voltage, 3500 V; nozzle voltage, 500 V; and fragmentor voltage, 120 V. The energies for collision-induced dissociation (CID) for fragmentation were set at 20 and 35 eV.

### Method validation

Linearity, limits of detection (LODs), limits of quantification (LOQs), repeatability, stability, intra-day precision and inter-day precision were assessed. A series of diluted mixed standard solutions was analyzed subsequently from low to high concentration for linearity, LODs and LOQs. The phloem of RGyueaj was selected for validating the method’s repeatability and stability. Repeatability was evaluated by six replicated analyses of the phloem at the similar locations in six tissue slices. Stability was tested on one sample solution at 0, 12, 24, 36, 48 h. Intra-day precision was performed by analyzing five replications of the mixed standard solution in 1 day while inter-day precision was examined by analyzing three replications of the solution in three consecutive days.

## Results and discussion

### Microscopic examination and dissection by LMD

**Fig. 2:**
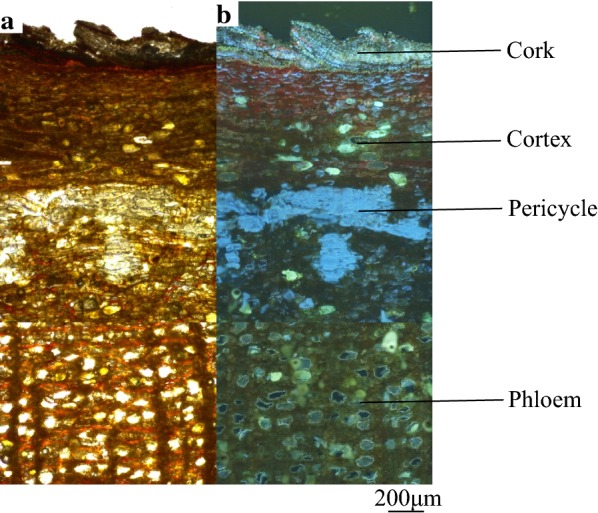
Microscopic characteristics of the *Cinnamomum cassia* (RGyueaj). **a** Observed under the light microscopy. **b** Observed under the fluorescent microscopy

As shown under the normal light and fluorescence mode (Fig. 2), the transverse section of Cinnamomi Cortex could be divided into four portions: cork (CK), cortex (C), pericycle (PE) and phloem (PH). Cork consists of several layers of cells and emits bluish-grey fluorescence. Cortex has a scattering of stone cells. Dark brown fluorescence was emitted from cortex to phloem, while a bright blue color was emitted from the pericycle. Pericycle was arranged in an interrupted ring. Phloem was broad with rays 1–2 rows of cells wide. Since different tissues possessed various features and could be distinguished under fluorescence mode, each separated tissue was dissected at the size of about 1,000,000 μm^2^ by LMD.

### Tissue-specific chemical profiling

**Fig. 3:**
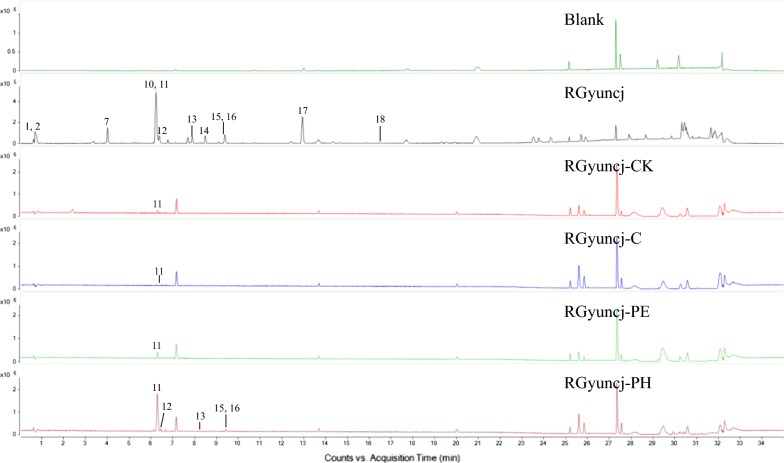
Representative UPLC-Q/TOF–MS base peak chromatograms of medicinal material sample and various tissues from *Cinnamomum cassia*

Tissue-specific chemical profiles were obtained as base peak chromatograms by UPLC-Q/TOF–MS (representative chromatograms are showed in Fig. 3). A total of 19 peaks were unequivocally or tentatively identified in the chromatogram of the medicinal material sample RGyuncj by comparing their retention times, m/z of molecular ions and/or fragment ions with standards or reported references [2, 11–16]. Five peaks were positively identified. Peaks 11, 13, 14, 15 and 16 were unambiguously identified as coumarin (147.0438 m/z, [M + H]^+^), cinnamic acid (149.0595 m/z, [M + H]^+^), cinnamaldehyde (133.0647 m/z, [M + H]^+^), cinnamyl alcohol (135.0802 m/z, [M + H]^+^) and 2-methoxycinnamaldehyde (163.0750 m/z, [M + H]^+^), respectively. 13 peaks were tentatively identified by comparison of their molecular ions of [M + H]^+^ or [M + Na]^+^ from literature reports. The detailed results are shown in Table 2.

**Table 2:** Chemical characterization of medicinal material sample of RGyuncj by UPLC-Q/TOF–MS

Peak no.	Identification	t_R_ (min)	Molecular formular	Measured mass (m/z)	Theoretical mass (m/z)	Mass accuracy (ppm)	Ion type	MS/MS (m/z)
1	Fructose^a^	0.71	C_6_H_12_O_6_	203.0522	203.0532	− 4.92	[M + Na]^+^	185[M+Na-H_2_O]^+^, 157[M+Na-CH_2_O_2_]^+^, 136[M+H-CHO_2_]^+^
2	Sucrose^a^	0.71	C_12_H_22_O_11_	365.1048	365.1060	− 3.29	[M + Na]^+^	351[M+Na-CH_2_]^+^, 203[M+Na-C_6_H_10_O_5_]^+^
3	(+)-Catechin^a^	3.33	C_15_H_14_O_6_	291.0856	291.0863	− 2.40	[M + H]^+^	185[M+H-C_3_H_6_O_4_]^+^, 123[M+H-C_12_H_8_O]^+^
4	Procyanidin B1 or B2^a^	3.34	C_30_H_26_O_12_	579.1484	579.1497	− 2.24	[M + H]^+^	409[M+H-C_8_H_10_O_4_]^+^, 309[M+H-C_9_H_18_O_9_]^+^, 123[M+H-C_27_H_19_O_7_]^+^
5	B-type procyanidin trimer^a^	3.92	C_45_H_38_O_18_	867.2116	867.2131	− 1.73	[M + H]^+^	579[M+H-C_13_H_20_O_7_]^+^, 439[M+H-C_16_H_28_O_13_]^+^, 377[M+H-C_17_H_30_O_16_]^+^, 344[M+H-C_18_H_35_O_17_]^+^, 123[M+H-C_42_H_31_O_13_]^+^
6	Procyanidin B1 or B2^a^	3.92	C_30_H_26_O_12_	579.1487	579.1497	− 1.73	[M + H]^+^	439[M+H-C_7_H_8_O_3_]^+^, 344[M+H-C_7_H_13_O_8_]^+^, 289[M+H-C_12_H_18_O_8_]^+^ 123[M+H-C_27_H_19_O_7_]^+^
7	B-type procyanidin tetramer^a^	4.10	C_60_H_50_O_24_	1155.2741	1155.2765	− 2.08	[M + H]^+^	867[M+H-C_8_H_18_O_9_]^+^, 579[M+H-C_22_H_40_O_17_]^+^, 483[M+H-C_45_H_20_O_7_]^+^, 351[M+H-C_46_H_28_O_14_]^+^, 171[M+H-C_52_H_40_O_20_]^+^
8	Cinnzeylanol^a^	4.67	C_20_H_32_O_7_	407.2037	407.2046	− 2.21	[M + Na]^+^	349[M+H-C_2_H_2_O_2_]^+^, 331[M+H-C_6_H_4_]^+^, 123[M+H-C_17_H_25_O_2_]^+^
9	Cinnacasside E^a^	5.20	C_25_H_38_O_11_	537.2297	537.2312	− 2.79	[M + Na]^+^	303[M+H-C_9_H_14_O_7_]^+^, 123[M+H-C_22_H_31_O_6_]^+^
10	Guiacol^a^	6.23	C_7_H_8_O_2_	147.0438	147.0422	10.88	[M + Na]^+^	118[M+Na-CHO]^+^, 103[M+Na-C_2_H_4_O]^+^
11	Coumarin^b^	6.23	C_9_H_6_O_2_	147.0438	147.0440	− 1.36	[M + H]^+^	103[M+H–CO_2_]^+^, 91[M+H-C_3_H_4_O]^+^, 77[M+H-C_3_H_2_O_2_]^+^ 65[M+H-C_4_H_2_O_2_]^+^
12	2-Hydroxycinnamaldehyde^a^	6.40	C_9_H_8_O_2_	149.0592	149.0597	− 3.35	[M + H]^+^	131[M+H-H_2_O]^+^, 121[M+H-CO]^+^, 103[M+H-CH_2_O_2_]^+^ 93[M+H-C_3_H_4_O]^+^, 91[M+H-C_2_H_2_O_2_]^+^, 77[M+H-C_3_H_4_O_2_]^+^ 65[M+H-C_4_H_4_O_2_]^+^, 55[M+H-C_5_H_2_O_2_]^+^
13	Cinnamic acid^b^	7.79	C_9_H_8_O_2_	149.0595	149.0597	− 1.34	[M + H]^+^	131[M+H-H_2_O]^+^, 123[M+H-C_2_H_2_]^+^, 103[M+H-CH_2_O_2_]^+^
14	(E)-Cinnamaldehyde^b^	8.28	C_9_H_8_O	133.0647	133.0648	− 0.75	[M + H]^+^	115[M+H-H_2_O]^+^, 105[M+H-CO]^+^, 103[M+H-CH_2_O]^+^ 91[M+H-C_2_H_2_O]^+^, 79[M+H-C_3_H_2_O]^+^, 77[M+H-C_3_H_4_O]^+^ 55[M+H-C_6_H_6_]^+^
15	Cinnamyl alcohol^b^	9.39	C_9_H_10_O	135.0802	135.0804	− 1.48	[M + H]^+^	117[M+H-H_2_O]^+^, 91[M+H-C_2_H_4_O]^+^, 55[M+H-C_6_H_8_]^+^
16	2-Methoxycinnamaldehyde^b^	9.39	C_10_H_10_O_2_	163.0750	163.0754	− 2.45	[M + H]^+^	145[M+H-H_2_O]^+^, 135[M+H-CO]^+^, 115[M+H-CH_5_O_2_]^+^ 107[M+H-C_3_H_4_O]^+^, 105[M+H-C_2_H_2_O_2_]^+^, 91[M+H-C_3_H_4_O_2_]^+^ 79[M+H-C_4_H_4_O_2_]^+^, 77[M+H-C_4_H_6_O_2_]^+^, 57[M+H-C_7_H_6_O]^+^ 55[M+H-C_7_H_8_O]^+^
17	Unknown	13.00	C_15_H_24_O_2_	237.1829	237.1849	− 8.43	[M + H]^+^	71[M+H-C_10_H_13_O_2_]^+^, 81[M+H-C_11_H_8_O]^+^, 89[M+H-C_10_H_12_O]^+^ 93[M+H-C_10_H_8_O]^+^, 105[M+H-C_9_H_8_O]^+^, 149[M + H-C_4_H_8_O_2_]^+^ 219[M+H-H_2_O]^+^
18	Dehydro-sesquiterpene oxide^a^	16.56	C_15_H_22_O	219.1741	219.1743	− 0.91	[M + H]^+^	150[M+H-C_4_H_5_O]^+^, 135[M+H-C_5_H_8_O]^+^, 121[M+H-C_6_H_10_O]^+^
19	Dehydro-sesquiterpene^a^	18.54	C_15_H_22_	203.1791	203.1794	− 1.48	[M + H]^+^	185[M+Na-C_3_H_5_]^+^, 150[M+H-C_4_H_5_]^+^, 136[M+H-C_5_H_7_]^+^ 123[M+H-C_6_H_8_]^+^, 103[M+H-C_7_H_16_]^+^

^a^Identified by previous literature reports

^b^Identified by standards

As seen from Table 3, peak 10 couldn’t be detected in any tissue of any sample. It can be assumed that the content of peak 10 is below LOD in herbal tissues. The totality of chemicals in cortex (5–12 peaks) and phloem (5–10 peaks) was slightly greater than those in cork (4–8 peaks) and pericycle (5–8 peaks). Peaks 11, 13, 14, 15, 16, namely coumarin, cinnamic acid, cinnamaldehyde, cinnamyl alcohol and 2-methoxycinnamaldehyde, could be detected in almost every tissue. Distinctly, the areas of these peaks were larger than those of other chemicals. Therefore, further quantitative analysis of them was carried out.

**Table 3:** The chromatographic peaks found in the chromatograms of each tissue in different specifications of cinnamon

Sample no.	Tissues/peak no. (T: totality)							
	CK	T	C	T	PE	T	PH	T
RGyueaj	1, 2, 11, 12, 13, 14, 15, 16	8	1, 2, 5, 9, 11, 12, 13, 14, 15, 16, 19	11	1, 2, 11, 13, 14, 15, 16	7	1, 2, 11, 13, 14, 15, 16	7
RGyuebj	1, 2, 11, 12, 13, 14, 15, 16	8	1, 2, 3, 4, 6, 9, 11, 13, 14, 16	10	1, 2, 4, 11, 14, 16	6	1, 2, 4, 9, 11, 13, 14, 15, 16	9
RGyuecj	1, 2, 11, 13, 14, 15, 16	7	1, 2, 4, 5, 7, 9, 11, 13, 14, 15, 16	11	1, 2, 11, 13, 14, 15, 16	7	1, 2, 11, 12, 13, 14, 15, 16	8
RGgxdxjcy	8, 11, 14, 16	4	2, 4, 8, 11, 13, 14	6	2, 8, 9, 11, 13, 14, 15, 16	8	2, 8, 11, 13, 14, 16	6
RGgxpnjcy	11, 13, 14, 15, 16	5	11, 13, 14, 15, 16	5	11, 13, 14, 15, 16	5	11, 13, 14, 15, 16	5
RGgddqjcy	11, 13, 14, 15, 16	5	11, 13, 14, 15, 16	5	11, 13, 14, 15, 16	5	11, 13, 14, 15, 16	5
RGgxdxzt	2, 11, 13, 14, 15, 16	6	2, 4, 6, 8, 11, 13, 14, 15, 16	9	2, 11, 13, 14, 15, 16	6	2, 11, 13, 14, 15, 16	6
RGgxpnzt	2, 11, 13, 14, 15, 16	6	2, 3, 5, 6, 8, 11, 13, 14, 15, 16	10	2, 11, 13, 14, 15, 16	6	2, 11, 13, 14, 15, 16	6
RGgddqzt	1, 11, 13, 14, 15, 16	6	1, 4, 5, 7, 8, 11, 13, 14, 15, 16	10	1, 2, 11, 13, 14, 15, 16	7	1, 2, 4, 5, 8, 11, 13, 14, 15, 16	10
RGyunaj	11, 13, 14, 15, 16	5	4, 5, 7, 11, 12, 13, 14, 15, 16	9	11, 13, 14, 15, 16	5	2, 11, 13, 14, 15, 16	6
RGyunbj	1, 4, 11, 13, 14, 15, 16	6	1, 4, 5, 11, 13, 14, 15, 16	8	1, 2, 11, 13, 14, 15, 16	7	1, 2, 11, 12, 13, 14, 15, 16	8
RGyuncj	1, 11, 13, 14, 15, 16	6	1, 2, 4, 5, 7, 8, 9, 11, 13, 14, 15, 16	12	1, 11, 12, 13, 14, 15, 16	7	1, 11, 12, 13, 14, 15, 16, 18	8
RGgxpnbg	11, 13, 14, 15, 16	5	11, 13, 14, 15, 16	5	11, 13, 14, 15, 16	5	11, 13, 14, 15, 16	5
RGgxdxbg	11, 12, 13, 14, 15, 16	6	11, 13, 14, 15, 16	5	11, 13, 14, 15, 16	5	11, 13, 14, 15, 16	5
RGlw	2, 8, 11, 12, 13, 14, 15, 16	8	2, 8, 9, 11, 12, 13, 14, 15, 16	9	2, 11, 12, 13, 14, 15, 16	7	1, 2, 8, 11, 13, 14, 15, 16	8
RGgxpngg	11, 13, 14, 15, 16	5	2, 4, 11, 13, 14, 15, 16	7	2, 11, 13, 14, 15, 16	6	2, 11, 13, 14, 15, 16	6

### Quantification of essential oils in various tissues

The results of method validation are presented in Table 4. The regression equation for each compound was calculated in the form of y = ax + b, where y and x were peak area and amount of compound injected, respectively. Each calibration curve possessed good linearity with correlation coefficients (r^2^) ≥ 0.9953 within the selected range. The LODs and LOQs were determined at signal-to-noise (S/N) ratios of 3 and 10, respectively. The repeatability ranged from 5.34 to 27.56%. The RSD value of stability was less than 11.66%, indicating that the stability of current method in this study was acceptable. The above assay results indicate that this developed method is reproducible, precise and sensitive enough for tissue-specific determination of five analytes in Cinnamomi Cortex.

**Table 4:** Method validation results

Analyte	Calibration curve	Linear range (ng/mL)	r^2^	LODs (ng/mL)	LOQs (ng/mL)	Repeatability (n = 6, RSD, %)	Stability (n = 5, RSD, %)	Precision RSD (%)	
								Intra-day (n = 5)	Inter-day (n = 3)
Coumarin	y = 905852x − 26008	51.525–1030.5	0.9981	19.1	56.1	17.43	5.99	3.17	2.81
Cinnamyl alcohol	y = 1486.4x − 350.23	267.6–11339	0.9970	29.0	147.3	27.56	2.03	6.13	32.66
Cinnamic acid	y = 66690x − 2038	66.35–1327	0.9982	159.3	334.2	5.34	7.34	4.31	5.27
Cinnamaldehyde	y = 539.3x + 833.7	2615.6–111058	0.9996	513.2	1053.0	10.37	3.40	2.45	30.50
2-Methoxycinnamaldehyde	y = 1*10^6^x − 5380.3	39.4–394	0.9953	9.3	52.7	9.26	11.66	23.97	28.40

**Fig. 4:**
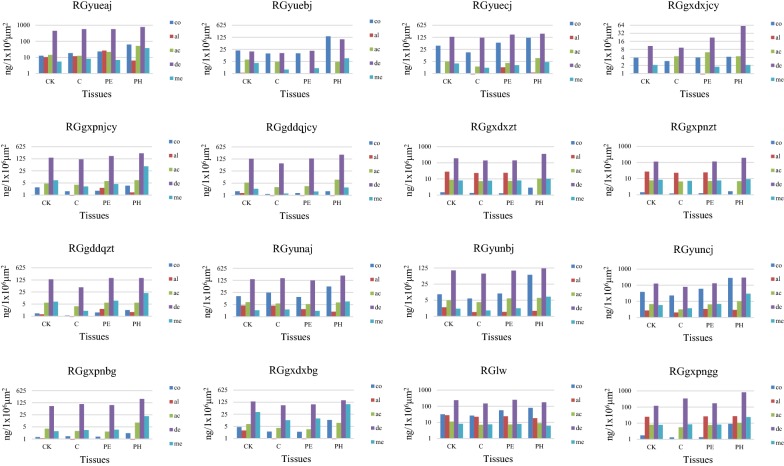
The contents of coumarin (co), cinnamyl alcohol (al), cinnamic acid (ac), 2-methoxycinnamaldehyde (me), cinnamaldehyde (de) in the tissue samples

The results of quantitative analysis (Additional file 1: Table S1 and Fig. 4) demonstrated that the content of cinnamaldehyde was much higher than other chemicals. Cinnamaldehyde was concentrated in phloem except for sample RGlw, where it was most abundant in the pericycle. 2-methoxycinnamaldehyde showed the same pattern of occurrence as cinnamaldehyde. Cinnamic acid was enriched in pericycle of sample RGgxdxjcy and in cork of samples RGgxpnzt and RGlw as well as in phloem of other samples. For all samples, phloem contained the highest amount of coumarin. Cinnamyl alcohol showed the highest content in phloem of one sample, in pericycle of six samples and in cork of others; thus, for this component, the pattern of distribution was difficult to determine. The irregularity may be due to its low content and/or its tendence to esterify easily.

## Conclusions

In the present study, an approach using LMD combined with UPLC-Q/TOF–MS was established to map the distribution of essential oils in tissues of various specifications of Cinnamomi Cortex. It is the first report with respect to tissue-specific metabolites in the cortex of an herb. This histochemical study identified Cinnamomi Cortex phloem as the tissue richest in essential oils. Thus, it would be logical to deduce that Cinnamomi Cortex with thick phloem is of better quality as it contains more active constituents. In fact, this is consistent with the traditional processing method of removing the outer bark. Our analytical method provides references for evaluating the quality and classifying the grades of Cinnamomi Cortex by thickness of phloem. Further studies can be conducted to explore the factors affecting phloem thickness. Therefore, this research can be of great importance in the cultivation, harvesting, processing and clinical application of Cinnamomi Cortex.

## Additional file

**Additional file 1: Table s1.** Contents of essential oils in various tissues of the samples.
