# Supplementary material for: Tissue-specific chemical profiling and quantitative analysis of bioactive components of Cinnamomum cassia by combining laser-microdissection with UPLC-Q/TOF–MS
Source: Chem Cent J. 2018 Jun 21;12:71. doi: 10.1186/s13065-018-0438-x (PMC6013415; doi:10.1186/s13065-018-0438-x)
Supplement: Supplementary file 1 — Additional file 1: Table s1. Contents of essential oils in various tissues of the samples. [file 13065_2018_438_MOESM1_ESM.docx]

Table S1 Contents of essential oils in various tissues of the samples

| Sample no. | Tissue | Amount in unit area (ng/10^6^μm^2^) | | | | |
| --- | --- | --- | --- | --- | --- | --- |
|  |  | Coumarin | Cinnamyl alcohol | Cinnamic acid | Cinnamaldehyde | 2-Methoxycinnamaldehyde |
| RGyueaj | CK | 12.92 | 10.54 | 14.50 | 453.12 | 5.58 |
|  | C | 18.83 | 11.98 | 12.73 | 578.01 | 8.32 |
|  | PE | 23.63 | 26.68 | 22.00 | 575.09 | 7.00 |
|  | PH | 63.58 | 6.39 | 52.25 | 772.31 | 38.07 |
| RGyuebj | CK | 21.65 | 1.11 | 6.46 | 18.79 | 4.10 |
|  | C | 14.51 | ND | 4.87 | 15.25 | 1.72 |
|  | PE | 15.03 | ND | ND | 20.81 | 2.03 |
|  | PH | 143.63 | 0.95 | 4.82 | 95.27 | 7.66 |
| RGyuecj | CK | 41.00 | 1.00 | 5.01 | 134.17 | 3.83 |
|  | C | 16.79 | 0.40 | 2.52 | 118.31 | 2.16 |
|  | PE | 60.60 | 2.31 | 4.10 | 179.42 | 3.04 |
|  | PH | 116.96 | 1.14 | 7.90 | 201.95 | 4.60 |
| RGgxdxjcy | CK | 3.92 | ND | ND | 10.70 | 2.10 |
|  | C | 2.92 | ND | 4.45 | 9.27 | ND |
|  | PE | 3.99 | 0.87 | 6.24 | 22.05 | 1.80 |
|  | PH | 4.23 | ND | 4.43 | 59.74 | 2.10 |
| RGgxpnjcy | CK | 2.79 | 0.95 | 4.62 | 143.53 | 7.12 |
|  | C | 1.65 | 0.58 | 3.92 | 117.02 | 3.13 |
|  | PE | 1.75 | 2.58 | 6.39 | 180.80 | 4.34 |
|  | PH | 3.48 | 1.42 | 7.16 | 260.91 | 45.70 |
| RGgddqjcy | CK | 1.67 | 1.32 | 5.37 | 130.18 | 2.38 |
|  | C | 1.16 | 0.39 | 2.93 | 69.19 | 1.26 |
|  | PE | 1.33 | 0.55 | 3.34 | 132.71 | 1.64 |
|  | PH | 1.68 | 0.49 | 7.91 | 221.75 | 2.82 |
| RGgxdxzt | CK | 1.47 | 28.14 | 8.66 | 189.16 | 7.98 |
|  | C | 1.34 | 23.48 | 7.22 | 138.45 | 7.73 |
|  | PE | 1.28 | 23.85 | 7.38 | 141.30 | 7.96 |
|  | PH | 2.87 | ND | 10.50 | 359.13 | 10.24 |
| RGgxpnzt | CK | 1.43 | 27.33 | 7.76 | 113.85 | 8.50 |
|  | C | 1.20 | 22.87 | 6.65 | ND | 7.28 |
|  | PE | 1.25 | 24.18 | 7.05 | 115.16 | 7.67 |
|  | PH | 1.64 | ND | 6.93 | 197.17 | 9.24 |
| RGgddqzt | CK | 1.46 | 1.29 | 6.09 | 135.89 | 6.85 |
|  | C | 1.11 | 0.38 | 3.77 | 47.15 | 2.04 |
|  | PE | 1.64 | 2.62 | 6.04 | 161.52 | 7.84 |
|  | PH | 2.27 | 1.73 | 6.05 | 161.11 | 21.58 |
| RGyunaj | CK | 15.24 | 4.40 | 6.87 | 146.07 | 2.36 |
|  | C | 24.68 | 4.28 | 5.79 | 168.22 | 2.63 |
|  | PE | 13.77 | 2.72 | 5.36 | 125.55 | 2.18 |
|  | PH | 55.02 | 1.94 | 6.58 | 238.26 | 7.55 |
| RGyunbj | CK | 9.00 | 2.48 | 4.94 | 98.60 | 2.15 |
|  | C | 5.97 | 1.54 | 4.10 | 71.75 | 1.79 |
|  | PE | 9.78 | 1.56 | 5.98 | 95.78 | 2.22 |
|  | PH | 63.21 | 1.73 | 6.31 | 119.47 | 7.07 |
| RGyuncj | CK | 38.78 | 2.72 | 6.74 | 126.53 | 5.88 |
|  | C | 23.10 | 2.05 | 3.16 | 78.33 | 3.72 |
|  | PE | 59.89 | 3.35 | 6.50 | 132.08 | 6.86 |
|  | PH | 284.31 | 2.95 | 10.03 | 299.18 | 29.68 |
| RGgxpnbg | CK | 1.29 | 1.11 | 3.95 | 79.71 | 2.81 |
|  | C | 1.43 | 0.88 | 2.88 | 103.49 | 3.26 |
|  | PE | 1.36 | 0.92 | 2.66 | 90.97 | 3.43 |
|  | PH | 2.17 | 0.42 | 8.88 | 206.48 | 20.74 |
| RGgxdxbg | CK | 4.52 | 2.89 | 6.74 | 135.83 | 33.54 |
|  | C | 2.48 | 0.43 | 3.99 | 82.48 | 11.29 |
|  | PE | 2.41 | 0.41 | 3.37 | 92.71 | 14.15 |
|  | PH | 11.55 | 0.69 | 7.90 | 160.99 | 95.55 |
| RGlw | CK | 31.83 | 27.81 | 11.15 | 236.89 | 8.24 |
|  | C | 26.09 | 22.26 | 7.05 | 147.99 | 7.47 |
|  | PE | 56.37 | 23.91 | 7.44 | 252.35 | 7.90 |
|  | PH | 78.66 | 18.07 | 8.89 | 176.48 | 6.23 |
| RGgxpngg | CK | 1.74 | 24.94 | 8.01 | 119.88 | 7.86 |
|  | C | 1.32 | ND | 5.55 | 340.68 | 8.38 |
|  | PE | 1.34 | 26.10 | 7.76 | 170.68 | 8.12 |
|  | PH | 9.04 | 27.01 | 10.66 | 816.74 | 23.73 |

ND: Not detected
